# Presenting an Approach for Conducting Knowledge Architecture within Large-Scale Organizations

**DOI:** 10.1371/journal.pone.0127005

**Published:** 2015-05-20

**Authors:** Touraj Varaee, Jafar Habibi, Ali Mohaghar

**Affiliations:** 1 Department of Computer Engineering, Science and Research Branch, Islamic Azad University, Tehran, Iran; 2 Department of Computer Engineering, Sharif University of Technology, Tehran, Iran; 3 Department of Management, Tehran University, Tehran, Iran; Tianjin University of Technology, CHINA

## Abstract

Knowledge architecture (KA) establishes the basic groundwork for the successful implementation of a short-term or long-term knowledge management (KM) program. An example of KA is the design of a prototype before a new vehicle is manufactured. Due to a transformation to large-scale organizations, the traditional architecture of organizations is undergoing fundamental changes. This paper explores the main strengths and weaknesses in the field of KA within large-scale organizations and provides a suitable methodology and supervising framework to overcome specific limitations. This objective was achieved by applying and updating the concepts from the Zachman information architectural framework and the information architectural methodology of enterprise architecture planning (EAP). The proposed solution may be beneficial for architects in knowledge-related areas to successfully accomplish KM within large-scale organizations. The research method is descriptive; its validity is confirmed by performing a case study and polling the opinions of KA experts.

## Introduction

Although many frameworks and methodologies have been proposed in the field of KM as depicted in [1], numerous enterprises have been unable to implement their KM projects despite large monetary investments [2]. Both technical factors and nontechnical factors are critical to the successful implementation of a KM project. Culture, KA, information technology infrastructure and supportive services are important factors in the successful establishment of a KM project [3, 4]. The relationship between KM and KA can be envisaged as follows: KM involves the production, presentation, storage, transfer and transformation of knowledge [5], whereas KA involves the extraction of knowledge and relevant knowledge managerial processes [6]. KA is required to ensure the successful implementation of a long-term or short-term KM project [7]. An example of KA is the design of a prototype before a new vehicle is manufactured.

Numerous large-scale organizations have emerged [8]. The system of these organizations is considered to belong to a ‘system of systems’ (SoS), which surpasses existing systems for the following reasons: the number of program code lines; the number of engaged individuals in the system; the stored, retrieved, manipulated and refined data; the rate of connectivity and the inter-unit dependence among software elements and hardware elements. Examples of these systems include global data grid networks, future telecommunication and communication networks, the Internet, electronic governments, and virtual cities. Large-scale organizations grapple with unresolved problems that require attention by engineers and scientists. One of these unresolved problems is knowledge architecting, which serves an undeniable role in promoting the likelihood of successful implementation and the modeling of KM projects.

Although different KA frameworks have been proposed, such as the frameworks proposed by Jafari [9], Lusa [10], and Zhang [11], none adequately fulfill the need for conducting KA within large-scale organizations. KA is a new discipline in which neither a universally accepted codified framework nor a methodology has been established. This paper addresses this shortcoming by reviewing previous KA methods and presenting a suitable methodology and supervising framework for conducting KA within large-scale organizations, which provides well-defined constructs and guiding principles to ensure that large-scale organizations do not deviate from a proper path of KA.

In the next section, the current literature and their insights are reviewed and analyzed. A method for conducting this research and the details of the proposed solution for KA within large-scale organizations will be provided in the Research Method and the Proposed Solution sections. After evaluating the proposed solution, the results of this study and recommendations for future studies will be discussed.

## Literature Review

Enterprise architecture is a program supported by frameworks that coordinates various fundamental aspects of an enterprise in a holistic manner. Its origin dates to 1987, when a new field emerged as a consequence of a paper published by Zachman [12]; this field was previously known as enterprise information architecture (EIA). An EIA relates organizational missions, goals, and objectives to business tasks, activities and relations and to the technology or IT infrastructures required for their execution. Although several EIA frameworks are directed at different communities, the Zachman framework [12] shapes the infrastructure for the majority of other EIAs. It describes a holistic model of an enterprise information infrastructure from six perspectives and six abstractions. The major drawback of the Zachman framework is the lack of any guidance on the sequence, process, or implementation of the framework. The EAP methodology [13] is a specific attempt to provide guidance for the implementation of the Zachman framework. The scope of this methodology consists of the first two lines of Zachman’s initial framework and the three columns associated with it. The methodology employs a business data-driven approach and has been presented in the form of a four-layered cake and seven phases.

Recently, there has been a growing interest in a class of complex and large organizations whose constituents are complex per se. One instance of these complex organizations is the large-scale ones [8] which have been applied in various fields including military, government, space, manufacturing, service industry, and aerospace. According to the literature review in this area, there are many trends for the successful development of large scale organizations, among which two being more prominent. The first is the evolution of the field of network and communication to provide an appropriate foundation for establishing large-scale organizations [14–17]. The second trend in recent years has been the ongoing effort to change enterprise architecting from classical to large-scale. Accordingly, the traditional architecture of organizations is undergoing fundamental changes as societies are transforming into a set of inter-connected networks. Table 1 highlights the deviations from classical architecting to large-scale architecting. Thus, these differences imply the contradiction of some features within traditional architecture and the necessity for a newer and more complex architecture for these types of organizations. Due to their novelty, the architectural frameworks proposed in the realm of large-scale organizations are so rare that only two frameworks were discovered after the identification of the proposed information architecture frameworks in the realm of large-scale organizations, which were consistent with the objectives of this paper, namely, Boxer et al [18] and Morganwalp et al [19].

**Table 1:** Large-scale architecting versus classical architecting [20].

	Large-Scale Architecting	Classical Architecting
**Architecting properties**	• Abstract, meta-level	• Domain specific systems level
	• Network-centric	• Several stakeholders
	• People-intensive	• Controlled development
	• Intensive communication infrastructure	• Static architecture
	• Network of various stakeholders	
	• Collaborative emergent development	
	• Dynamic architecture	
**Architecting constraints**	• The same classical system architecting processes, but at the meta-level	• Architecting processes at component and systems levels
	• Scalability	• Monolithic system architecting (optimization of individual systems)
	• Interoperability	• Clear life cycle context
	• Trustworthiness	
	• Hidden cascading failures	
	• Confusing life cycle context	

**Fig 1:**
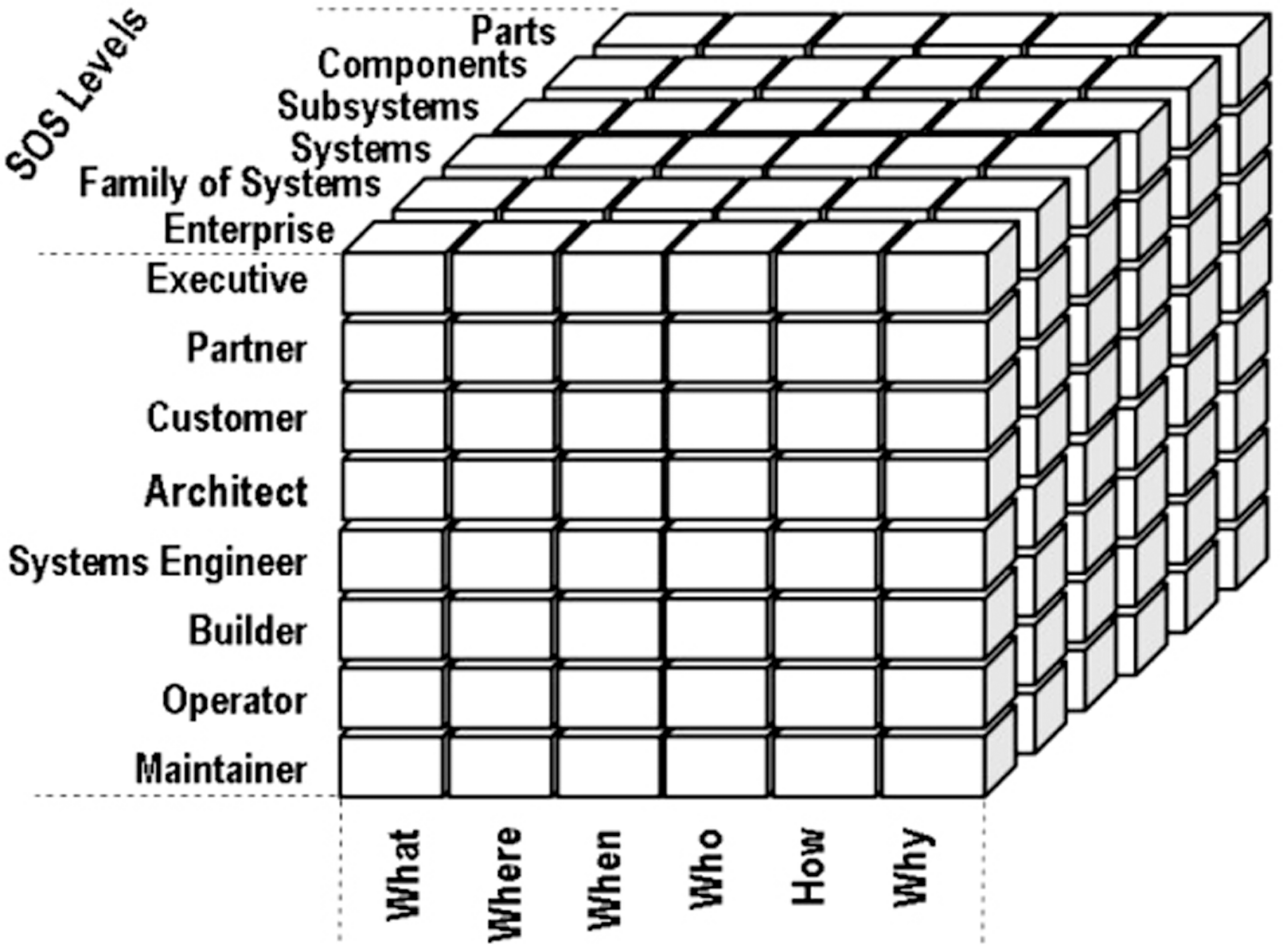
3D EAF proposed by Morganwalp [19].

From the perspective of Boxer et al, the main function of large-scale information architecture is interoperation among enterprise processes to promote enterprise values. Within the framework proposed by Morganwalp and his colleagues, namely, the Three-Dimensional Enterprise Architectural Framework or 3D EAF (Fig 1), a third dimension has been added to the Zachman’s two-dimensional information architecture framework [12] to show the level of architectural details within large-scale organizations. As a result and based on the conducted literature review, the interoperability and architecture level dimension are the minimum requirements that an architecture framework should provide in the realm of large-scale settings.

**Fig 2:**
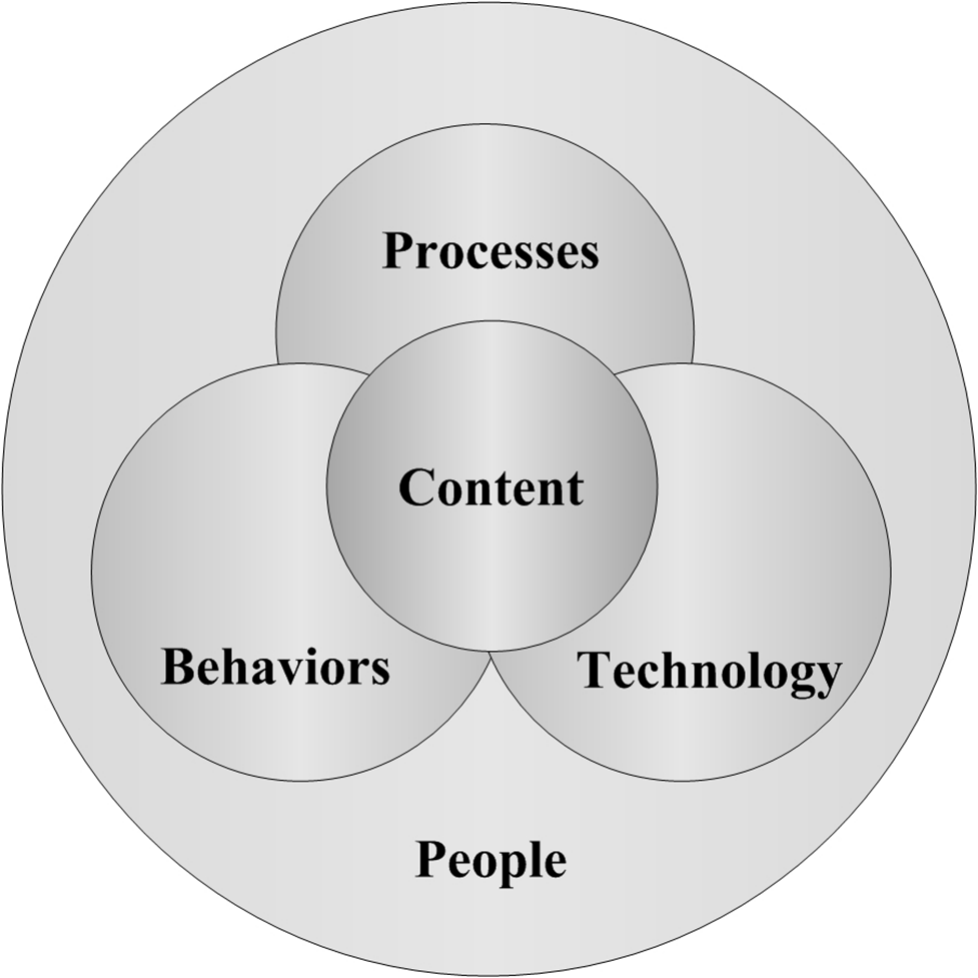
Componential elements of knowledge architecture [25].

A review of the parameters that were effective in the operationalization of a KM enterprise yields four factors: culture, KA, IT infrastructure and supportive services [3, 4]. The relationship between KM and KA can be envisaged as follows: KM involves the production, presentation, storage, transfer and transformation of knowledge [5], whereas KA involves the extraction of knowledge and KM processes [6]. The existence of KA has always been necessary for ensuring the successful operationalization of a long-term or short-term KM project [7]. The acquisition and application of explicit knowledge for gaining success in competitive markets requires an organized framework that is referred to as ‘knowledge architecture’ [21]; KA is contingent upon the concept of knowledge [22, 23]. Knowledge reservoirs and flows are considered to be the main elements in the formation of KA in an enterprise. Thus, KA can be assumed to be a component of enterprise assets [21]. Despite numerous definitions of KA that have been created by researchers in this field, the definition proposed by Lasnik seems to be more generic. He suggests that [24] “KA expounds the place and the manner of extracting and transferring enterprise knowledge. This type of architecture includes both explicit and implicit knowledge and is also designed for a providing thorough support for information and business architecture. In other words, KA incorporates the manner of creating knowledge, its application and learning within enterprise.” Therefore, the comprising elements of a KA could be defined as follows [7, 21] (Fig 2): people (employees of an organization, especially the staff of a knowledge department, the authors and the knowledge holders), processes (used by the employees of a knowledge department to achieve organizational goals and missions), behaviors (the behaviors of the employees of a knowledge department in a setting in which the processes of knowledge management must occur), technology (information technology, which facilitates the detection, creation and sharing of knowledge among the elements inside and outside an organization) and content (a shared knowledge database that has been electronically extracted).

A common engineering methodology in the scope of KM is CommonKADS [26]. CommonKADS is a comprehensive methodology which addresses all KM processes within an integrated and comprehensive structure [27]. From the viewpoint of CommonKADS, knowledge engineering is a process during which different models are created and each aspect of an individual’s knowledge can be recounted. These models are categorized into three separate categories: context category, concept category, and artifact category. Identifying and perceiving the domain of enterprises and the conceptual explanation of the knowledge applied in knowledge-based tasks in enterprises are the most important objectives of contextual and conceptual models, respectively. The artifactual models address the technical aspects and the materialization of KM though computerized systems.

Despite an extensive literature review by the authors of this paper, no comprehensive methodology was identified within the field of KA; the only findings in this filed referred to two brief studies of the KA framework performed by Jafari [9] and Lusa [10]. The knowledge architecture framework proposed by Jafari [9] is one of the initial frameworks in this area. They used the Zachman information architecture framework to develop their knowledge architecture framework. In another study by Lusa [10], the TOGAF information architecture framework was employed as the basic framework. The lack of a specific purpose and large-scale aspects for defining a framework are among the main problems that will be considered while exploring these frameworks.

With regard to the literature review in the field of KA within large-scale organizations, the following limitations comprise the main shortcomings in this field:

- **Deficiency in addressing properties of large-scale architecting:** With regard to the significant differences between large-scale architecting and classical architecting, the authors were not able to obtain knowledge architecture methodologies and frameworks that address the properties of large-scale architecting in the available literature.
- **Lack of a methodology that provides a suitable level of details:** After exploring the level of details stipulated in the KA development process, we conclude that some development processes should be referred to as frameworks instead of methodologies due to their lack of detail.
- **Lack of a supervising framework:** The field of KA lacks a suitable methodology that employs a framework as an initial structure for its methodology.

Therefore, this paper presents a solution for overcoming these limitations and responding to the following research question: “What type of methodology and supervising framework can be compiled using enterprise information architecture concepts for conducting KA within large-scale organizations?”

## Research Method

The descriptive method of research was employed due to the fundamental and qualitative nature of the objective of this research, which was the development of a methodology and a supervising framework for conducting KA within large-scale organizations. This method was selected due to its ability to pinpoint knowledge architecture via theoretical, genuine, contextual and comparative modeling and utilize knowledge management and information architecture procedures. The corpus of the study included the scientific documents, papers and books that were relevant to assess the validity of the proposed solution regarding the subjective and objective aspects, the following assessment criteria were employed after conducting extensive investigations. We employed questionnaires to collect the viewpoints of elite experts and personnel in KM and KA and the Delphi technique [28] as the consensus methodology. To select the elite experts and personnel who form our Delphi population, a combination of judgmental and snowball sampling [29] was applied:

- Assessment via a case study and an appropriate KM capability maturity model to evaluate the effect of the proposed KA methodology and the framework in the study organization. For this reason, information about all knowledge-intensive inventories, such as leadership, culture and structure, processes, explicit knowledge, implicit knowledge, knowledge hubs and centers, market leverage, measures, employee skills and technological infrastructure, was collected from the study organization before and after implementing the KA and employed during the assessment process.
- Assessment using qualitative methods and expert viewpoints about the proposed KA methodology and framework.

We confirm that the written consent of all participants in this study, including the experts and the faculty members, was obtained; furthermore, the whole study including the questionnaires and the case study, was reviewed and approved by the review board of the Ports and Maritime organization in written consent form with the 1390–7242 approval number prior to the study.

## Proposed Solution

Our solution addresses the shortcomings that were identified in the literature review section. In the first step, we present a KA framework within large-scale organizations. This framework contains a set of principles, patterns and guidelines at the macro level that can be used to conduct KA within large-scale organizations and to address the lack of a supervising KA framework. In the second step, while acknowledging the principles and structures specified in the proposed framework, we present a methodology for conducting KA that addresses large-scale architecting and possesses a sufficient level of detail to enable a practical and executable process for KA in these settings. Our proposed methodology and supervising framework for conducting KA within large-scale organizations will be addressed in the following section using two steps.

### 1. The proposed framework

From the literature review in this area of research [9, 10], we determined that the first step in presenting a knowledge architecture framework (KAF) is the selection of an enterprise information architecture framework (EIAF) as the basic framework for KA. Consequently, the researchers decided to employ Zachman’s EIAF. This framework was selected as the basic framework for re-writing and application in the area of KA for the following reason:

- Serves as the only accepted EIAF and the de-facto standard in the area of information architecture and shapes the infrastructure for the majority of other EIAFs, such as the FEAF, FEA, and the TEAF.
- Independently and simultaneously focuses on different aspects
- Preserves the integrity of different models and addresses all required models for describing architecture within an enterprise
- Possesses simplicity and proper integrity
- Exhibits a formal and robust structure

After the selection of a basic EIAF, the next step involves responding to this question: How can Zachman’s EIAF be indigenized for admitting KA’s products to ensure that the rules governing Zachman’s framework [30] are not violated and to ensure that the resulting framework simultaneously encompasses all requirements of KA. The idea of transforming the fields of all cells within Zachman’s framework from information into knowledge creates a KAF within non-large-scale organizations. In this method, the content of each cell is proportional to the nature of the cell (perspective and the abstraction attributed to it) and is consistent with KA. This framework exhibits two dimensions: the first dimension incorporates the notion that each of these perspectives represents one of the main participants in the preparation of KA, which is congruent with the perspectives within Zachman’s framework. The second dimension also describes different abstractions for KA, which correspond with the abstractions within Zachman’s EIAF.

As mentioned in the literature review, interoperability and architecture-level dimensions are the minimum requirements that an architecture framework should provide in the realm of a large-scale setting. Numerous methods have been proposed for enacting interoperation within this set of organizations [31]. Service-oriented methods seem to be more appropriate by virtue of the type of attitude toward distribution. In service-oriented KA within large-scale organizations, service-oriented architecture is a style of KA that is dependent on a service-oriented outlook in its models and artifacts for re-learning KA. The addition of a new abstraction is the best option for enacting service-oriented methods within our proposed KAF. Executing this method compensates the non-integrity and lack of connection among different abstractions using service-oriented architectural styles and the promotion of a KAF from categorizing models and documents of KA to their integrator. The basic model of the new abstraction, which is referred to as service, is defined as the service-contract-receiver, in which the service receiver encompasses any virtual existences (a service, a program or a permitted person).

**Fig 3:**
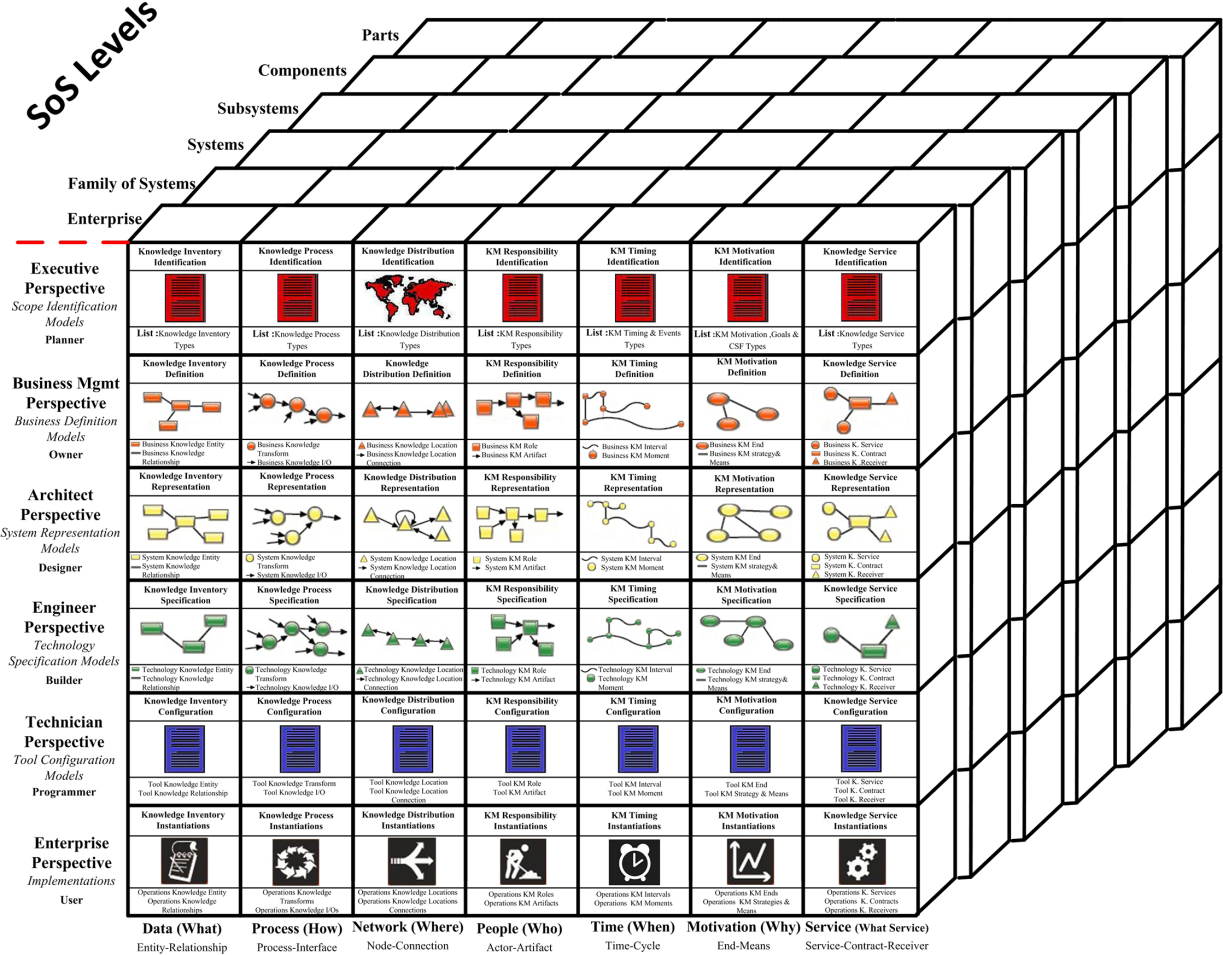
Proposed KAF for large-scale organizations.

The 3D framework in Fig 3 can be considered to be the final version of our proposed KAF within large-scale organizations. It was created by adding a new architecture-level dimension entitled “SoS Level” and service abstraction to Zachman’s EIAF and changing the scope of all cells from information to knowledge. The architecture-level dimension provides a group of architectures; each architecture supplies different business perspectives by varying the level of details and addressing related but distinct concerns. Large-scale organizations and the different viewpoints of each type of architecture are hierarchically organized. The three dimensions within our proposed framework are as follows:

- **Abstraction** comprises the abstractions of data, process, network, people, time, motivation and service.
- **Individuals’ perspectives** comprise the viewpoints of the planner, the owner, the designer, the builder, the programmer and the user.
- **Hierarchical levels** comprise a hierarchical knowledge architecture that includes different levels, such as enterprise, family of systems, systems, subsystems, components, and parts.

All cells in the framework have been re-written and updated in proportion with the requirements of KA and have changed their scope from information to knowledge. Because our objective is to provide a version of KAF, we will only concentrate on the first three lines of the framework and consider the fourth, fifth and the sixth lines to exceed the boundary of KAF. The contents of each cell in the first three perspectives of the framework are described as follows:

- **Planner’s perspective (executive perspective):**
  - **Data abstraction:** This cell contains a document that describes the knowledge inventories, such as entities, culture, and knowledge areas, and the enterprise value from macro and strategic viewpoints.
  - **Process abstraction:** This cell contains a document that describes the knowledge processes and knowledge entities that are engaged with these processes and the enterprise value from macro and strategic viewpoints.
  - **Network abstraction:** This cell contains a document that describes the macro-points for the geography of the production, distribution and consumption of knowledge in business.
  - **People abstraction:** This cell contains a document that explains the responsibilities of all participants engaged in enterprise knowledge management on a macro basis, including owners, producers, distributors and consumers.
  - **Time abstraction:** This cell contains a list of KM timing and events that provide knowledge from a business perspective and should be considered by an enterprise.
  - **Motivation abstraction:** This cell includes a list of objectives and goals of an enterprise for operationalizing KM within the enterprise. In addition, it notes the critical factors for the success of operationalizing KM strategies within an enterprise.
  - **Service abstraction:** This cell contains a document that describes the services related to business knowledge and consumer’s existences that are connected with these knowledge services from macro and strategic viewpoints.
- **Owner’s perspective (business management perspective):**
  - **Data abstraction:** This cell describes the business entities and their interrelations from the perspective of a planner and presents a proper classification for these business entities.
  - **Process abstraction:** This cell defines each of the macro knowledge processes from a planner’s perspective, which comprise other business knowledge processes and resources from the perspective of high-level and middle-level managers.
  - **Network abstraction:** By relying on the viewpoints of high-level and middle-level managers, this cell defines the ‘how’ model for the relation among geographical situations of business producers, distributors and consumers of knowledge, which have been identified from the planner’s perspective.
  - **People abstraction:** This cell contains the business responsibilities that are specific to employees and knowledge authors and the description of their artifacts. This cell defines the business knowledge roles, artifacts and knowledge-centered enterprise positions within an enterprise’s structure.
  - **Time abstraction:** This cell contains a description of business the KM life cycle within an enterprise and all related business KM intervals and moments.
  - **Motivation abstraction:** This cell describes the business ends, motivational strategies, and means that can be applied for progressing KM operationalization within an enterprise; they correspond to the major objectives and strategies of KM within an enterprise.
  - **Service abstraction:** This cell defines and describes business knowledge services to form the known knowledge services from a planner’s perspective, according to high-level and middle-level managers’ viewpoints, and provides a comprehensive description for each service via a complete delineation of service functionalities, inputs, outputs, preconditions, post-conditions and authorized consumers.
- **Designer’s perspective (architect’s perspective):**
  - **Data abstraction:** Within this cell, the models for mapping the business knowledge entities to the system knowledge entities are defined by considering the knowledge entities and the identified relations from an owner’s perspective. Knowledge architecture prepares and designs the logical model for knowledge bases, which is required for storing enterprise knowledge by addressing system knowledge entities and relations.
  - **Process abstraction:** Within this cell, a knowledge architect can prepare the functionalities for different parts of a KM system (KMS) in accordance with the models for identifying, extracting, storing, sharing and applying knowledge and presents the architectural model for KMS.
  - **Network abstraction:** Within this cell, the logical network model for the distribution of knowledge is prepared and designed by considering the logistical network of the knowledge identified from the owner’s perspective, the enterprise knowledge items and the entities.
  - **People abstraction:** Within this cell, a knowledge architect prepares and designs the logical model of interfaces and the different roles and levels of access to each interface for the KMS.
  - **Time abstraction:** Within this cell, a knowledge architect designs the model for the sequence of system knowledge management intervals and moments and the manner of knowledge transformation from one type to another type within the KMS and explicates the method for mapping the mentioned sequence using system and business knowledge processes within an enterprise.
  - **Motivation abstraction:** This cell describes the business rules and logics for KM within an enterprise, which should be designed as preserved information and suitable structural and operational announcements within KMS. These business rules and logics occur in data structures (first column), description processes (second processes) and executive policies within different abstractions related to enterprise knowledge.
  - **Service abstraction:** Regarding business knowledge services, contracts, and receivers that are detected from the owner’s perspective, the mapping models for converting business services to systematic services are prepared in this cell. A knowledge architect prepares and designs the architectural model for presentable systematic knowledge services by focusing on the knowledge services presented from the side of enterprise to all authorized service consumers. This architectural model contains the schematic-structural definitions for knowledge services, structure and manner of communicating messages and the components required for each systematic knowledge service.

### 2. The proposed methodology

A well-defined and comprehensive KA framework that does not contain a process for development would be useless because the framework is not utilized. We attempt to identify a proper EIA methodology that is congruent with Zachman’s framework because Zachman’s EIAF was employed as the basis for the proposed KAF. In the second step, we attempt to re-write and update the selected methodology with regard to the KA approach and in accordance with the proposed KAF. Each of these two steps is explained in the next section.

From the authors’ viewpoint, the EAP methodology [13] is the only methodology that can be employed to develop and present our ideal KAF for the following reasons:

- The only methodology for conducting EIA according to Zachman’s framework
- Zachman’s EIAF as the basis for the proposed KAF
- Simplicity, well-defined nature and superiority over other methods and for conducting EIA in different enterprises

The best approach to presenting our KA methodology based on the EAP methodology is the initial structure of EAP in conjunction with its adaptation and upgrading to completely address the issue of KA within large-scale organizations and simultaneously employ the intended KA as a supervising framework. To update the scope of the EAP from information to knowledge, we have reused some models that are associated with the CommonKADS [26] knowledge engineering methodology. Because the domain of the work for our proposed KAF comprises three perspectives and the models presented in the contextual and conceptual categories of the CommonKADS methodology are consistent with the objectives of these three perspectives, the best option is reuse of the models in these two categories for upgrading the scope of the EAP methodology from information to knowledge.

**Fig 4:**
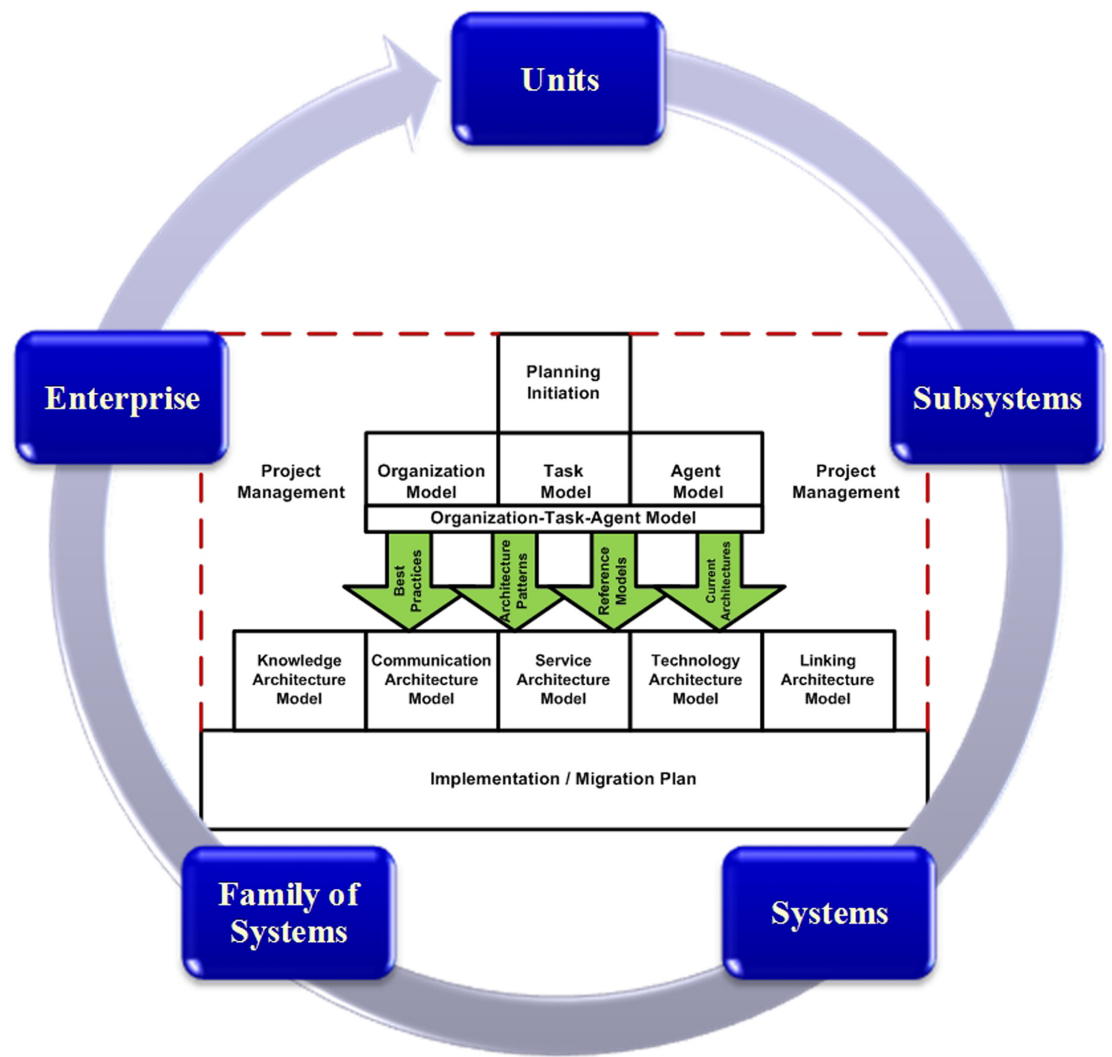
Proposed KA methodology for large-scale organizations.

Fig 4 illustrates our proposed methodology for conducting KA within large-scale organizations. Its domain and scope address the first three perspectives and the seven abstractions within the proposed KAF. The 4th, 5th and 6th perspectives of the framework have not been addressed by the methodology because they are beyond the objectives of KA. The iteration and evolution cycle of the methodology alludes to the architecture-level dimension of the proposed KAF. It states that as large-scale organizations are themselves hierarchically organized, the process of creating KA within these organizations occurs through iteration and evolution within the architectural models of the sub-organizations. The methodology has been compiled using five layers and 12 phases; each phase consists of some executive steps and outputs. These outputs sometimes exist as documents and results or as an architectural model, which are distinctively related to one of the cells within the framework and are frequently referred to as artifacts.

The first layer contains a phase that is referred to as “planning initiation”, which is the preplanning phase that is critical to setting all things in place, which enables the use of the proposed methodology to create a KA. Table 2 explicates all proposed phases, executive steps and outputs for this layer.

**Table 2:** Detailed description of the first layer of the proposed KA methodology.

Phase	Executive steps	Output
Planning Initiation	✓Provide KM vision document	• KM vision document
	✓Define the scope and domain of KA	• Documentation of scope and domain of KA
	✓Define the architecture levels that comprise a large-scale organization	• Documentation of project resources
	✓Adapt the process of developing KA	• KA methodology handbook
	✓Define and select the required resources for developing KA	• Documentation of project structure, the duties for each role and the work manual for each member
	✓Form the KA team	• The Gant chart for project execution
	✓Prepare the work schedule for KA	• Documentation of management’s verifications
	✓Receive management verification	

The objective of the second layer is to clarify the as-is knowledge architecture. It consists of four phases: “Identification of organization model”, “Identification of task model”, “Identification of agent model” and “Identification of organization-task-agent model”. The organization model is used to analyze an organization and shows how a knowledge-related solution affects an organizational structure and establishes feasibility and bottlenecks. The task model serves as a link between a human task and an organizational task. It describes a goal-oriented and knowledge-intensive activity with some value to an organization. The agent model elaborates which knowledge task is being performed by a particular agent. Although an organization-task-agent model is not directly involved in the development of knowledge architecture, it is used in managerial decision-making about changes, improvements in an organization, changes in a task or changes in an agent for a task. Table 3 explains all proposed phases, executive steps and outputs for this layer.

**Table 3:** Detailed description of the second layer of the proposed KA methodology.

Phase	Executive steps	Output
Identification of organization model	✓Prepare, schedule and conduct interviews	• Organization Model -1^a^
	✓Identify the problems, solutions and organizational domain	• Organization Model -2^a^
	✓Explain the organization focus from various points of view: structure, process, personnel, resources, knowledge, culture and capabilities	• Organization Model -3^a^
	✓Identify the main tasks of an organization business process	• Organization Model -4^a^
	✓Identify the knowledge resources within the organization business domain	• Organization Model -5^a^
	✓Prepare the feasibility study document	
Identification of task model	✓Analyze the tasks within the organization from various points of view: data, operation, knowledge intensive, describing internal or external duties of organization	• Task Model -1^a^
	✓Identify and explain knowledge resources	• Task Model -2^a^
Identification of agent model	✓Re-organize the information from the viewpoints of agents engaged in responsibilities	• Agent Model-1^a^
Identification of organization-task-agent model	✓Feasibility study, summary of the changes, improvements and proposed measures for enacting KA within the organization and its effects	• Organization-Task-Agent Model-1^a^

^a^These artifacts have been obtained from the artifacts in the context category of the CommonKADS methodology[26]

The third layer clarifies the to-be knowledge architecture situation and consists of five different phases: “Identification of knowledge architecture model”, “Identification of communication architecture model”, “Identification of service architecture model”, “Identification of technology architecture model”, and “Identification of linking architecture model”.

The knowledge architecture model is a tool that can be used to clarify the structure of a knowledge intensive information processing task. The communication model specifies information and knowledge exchange among different knowledge agents. The development of a process for service-oriented analysis, the extraction of atomic and composed knowledge services within an architecture area and the logical categorization of knowledge services based on their operations are among the most important goals of the service architecture model. The goal of the technology architecture model is to identify the standards, topologies and technologies that are required for implementing KA within an organization. Mapping, integrating and distributing KA models are the objectives of the linking architecture model. Table 4 clarifies all proposed phases, executive steps and outputs for this layer.

**Table 4:** Detailed description of the third layer of the proposed KA methodology.

Phase	Executive steps	Output
Identification of knowledge architecture model	✓Knowledge identification, which includes identification of the knowledge items in the domain and the process of organization	• Task Knowledge ^a^
	✓Knowledge specification, which includes creation of the initial domain scheme and completion of the organization knowledge specification models that contain the domain knowledge model, the task knowledge model and the inference knowledge model	• Inference Knowledge ^a^
	✓Knowledge refinement, which includes development of the knowledge base and the validation of the knowledge model	• Domain Knowledge ^a^
Identification of communication architecture model	✓Compile the communication plan, which includes a dialogue diagram and control over the transactions model	• Communication Model-1 ^a^
	✓Compile the transactions description model	• Communication Model-2 ^a^
	✓Compile the information exchange specification model	
	✓Define the candidature business and software services	
Identification of service architecture model	✓Transform the procedural model into re-usable components	• The architecture model of the procedural knowledge services layer
	✓Identify and define the architecture model of the procedural knowledge services layer	• The architectural model of the business knowledge services layer
	✓Identify and define the architecture model of the business knowledge services layer	• The architectural model of the software knowledge services layer
	✓Identify and define the architecture model of the software knowledge services layer	
	✓Identify and define the architectural distribution model for the knowledge service layers	
Identification of technology architecture model	✓Identify the standards and technologies	• Documentation of candidate platforms and tools for data bases, systems and networks
	✓Identify and define the network topology and the network architecture	• Documentation of network architecture
	✓Identify and define the users of systems	• Documentation of architecture for groups and levels of access for users
	✓Identify and define the technology architecture distribution model	
Identification of linking architecture model	✓Map the architectures	• Documentation of macro model of relations among architectures
	✓Integration of architectures	
	✓Distribute linking architectures model	

^a^These artifacts have been obtained from the artifacts in the concept category of the CommonKADS methodology[26]

The fourth layer contains a phase that is referred to as the “implementation/migration plan”. This plan is the outcome of the analysis of the gap between the as-is and to-be knowledge architectures. As an outcome of this step, the organization will be ready to begin transition to the to-be architecture via an organized and prioritized plan. Table 5 explains all proposed phases, executive steps and outputs for this layer.

**Table 5:** Detailed description of the fourth layer of the proposed KA methodology.

Phase	Executive steps	Output
Defining the transition plan	✓Identifying the sequence of required knowledge systems and the executive actions for implementation,	• Documentation of the list and the specification of the required knowledge systems
	✓Estimation of the resources	• Documentation of the estimation for the required resources, profit and cost related to the transition plan
	✓Preparation of a clear time plan	• Documentation of the required training programs
	✓Estimation of the profit and cost for the transition plan	
	✓Finalization of the key success factors of the project and development of suggestions	

The fifth layer is parallel with all other layers and contains one phase, which is referred to as “project management”. Each activity in any scope within organization will be managed as a project that has a schedule, a work breakdown structure and specific deliverables. Thus, knowledge architecture activity is not exempt from this rule, and this layer attempts to address this need. Table 6 explicates all proposed phases, executive steps and outputs for this layer.

**Table 6:** Detailed description of the fifth layer of the proposed KA methodology.

Phase	Executive steps	Output
Defining the compiling KA project management	✓Creation of project management plan, including	• Project Foundation ^a^
	– The life cycle of project, goals, and domain	• Financial Management ^a^
	– The project’s presentable artifacts	• Schedule Management ^a^
	– The work breakdown structure of the project	• Resource Management ^a^
	– The project schedule	• Communication Management ^a^
	– The existing resources that correspond to the stipulated budget	• Issue Management ^a^
	– The structure of the project team	• Risk Management ^a^
	– The manner of interaction among individuals inside and outside of the project	• Scope Change Control Management ^a^
	– Required experiences and training for team members	
	✓Create the quality plan of the project	
	✓Document the project management artifacts in each cycle	
	✓Assess the risks of the project	
	✓Provide the reports or periodical sessions for announcing the development of the KA compilation project	
	✓Provide the report for assessing the termination of project and the results	

^a^These artifacts have been obtained from the artifacts in the PRINCE2 project management methodology [32]

**Fig 5:**
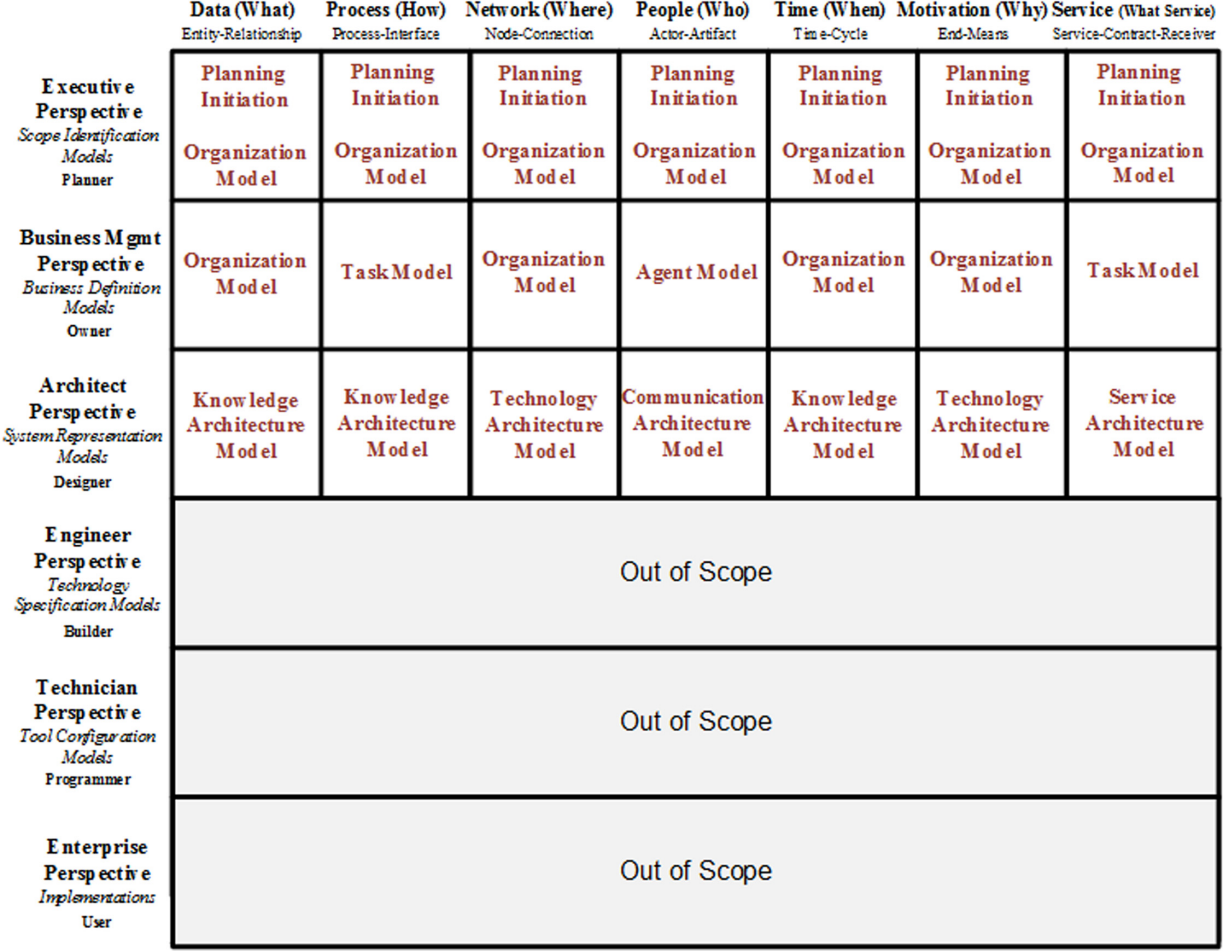
How the cells of the proposed KA framework are addressed by KA methodology models.

To demonstrate how the cells within the proposed KA framework are addressed by methodology models, we employed Fig 5. The 4th, 5th and 6th perspectives are placed outside of the issue of the methodology for conducting KA to concentrate on knowledge systems implementation. Conversely, the first perspective of the framework has been addressed by the models within the as-is layer, the second perspective has been addressed by the models within the to-be layer and the third perspective has been addressed by the models within the third layer of the proposed methodology.

## Evaluating the Proposed Solution

The proposed solutions within the field of architecture are less verifiable through formal methods. Thus, after extensive investigations and a review of different methods of assessment within this field, the researchers decided to employ a case study and qualitative assessment methods to examine the validity of the proposed solution regarding its subjective and objective aspects. In the next section, the assessments conducted by each of these two methods will be examined.

### 1. Assessment via a case study

The solutions presented in the field of architecture are commonly assessed by performing a case study. Note that a case study is an initial assessment that is performed within a controlled experimental environment. As a result, quantitative and precise confirmation of the advantages and strengths of a solution within the field of architecture is not possible. As a result, the solutions proposed by architects in different projects are necessary to develop a reliable assessment of the results.

Our case study was conducted in three stages: selecting an appropriate organization, producing artifacts according to the proposed solution and using a suitable KM capability maturity model for assessing the level of organization KM maturity under study. With regard to selecting the research organization, the researchers were compelled to restrict and reduce the scope of the study for two reasons: first, this study required access to a large-scale organization, such as a virtual city, for a real-world assessment; second, researchers were limited by time, human resources and mandates for conducting the case study. This restriction would not causes any distortion of the validity of the conducted assessments due to the features of iteration and the evolution KA development strategy within the proposed methodology and the architecture-level dimension within the proposed framework. We will generalize the results of this case study for an entire organization.

**Fig 6:**
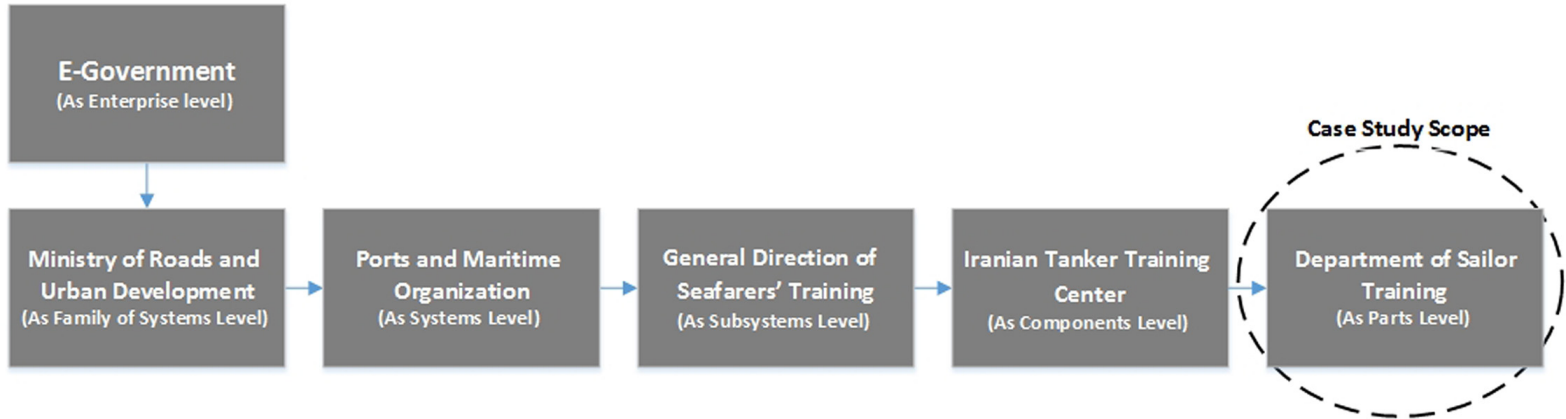
Diagram for architectural levels of large-scale organization in the case study.

The electronic government was initially selected as the large-scale organization due to its features. Based on the identification of the dimensions related to this organization according to Fig 6, the Sailor Training Department within the Iranian Tanker Training Center was specified as the area for the case study. By collecting the data within this organization and implementing the guidelines given by the proposed methodology, the KA for the as-is and to-be situation was extracted in the form of the artifacts of the proposed framework. We employed the KM capability maturity model of the K-Business Readiness Assessment, which was developed by Skyrme [33] to assess the level of research organization KM maturity after implementing the KA that emanated from the proposed solution. Access to the details of the model, the potential use of the model for different organizations, and the development of additional criteria for assessing an organization and the applicability and tangibility of the questions within this model for experts in non-KA fields explain why the Skyrme KM capability maturity model was selected.

A group of ten experts were employed to assess the level of KM maturity within the study organization. These experts had managerial experience in educational departments and possessed Ph.D. and master’s degrees. We asked them to identify the levels of ten metrics for the organization by completing the questionnaire based on the Skyrme model before and after implementing the KA plan. The ten metrics included leadership, culture and structure, processes, explicit knowledge, implicit knowledge, knowledge hubs and centers, market leverage, measures, personnel skills and technological infrastructure. Throughout this process, prior to completing the questionnaires and to improve the response quality of the participants, we requested their participation in a two-hour meeting titled “A Review over the Concepts of KM”. We employed Cronbach’s alpha to confirm the reliability of the questionnaire, which showed a suitable reliability value of 0.871 for this questionnaire using the SPSS software, version 21 and the Delphi technique as the consensus methodology.

**Fig 7:**
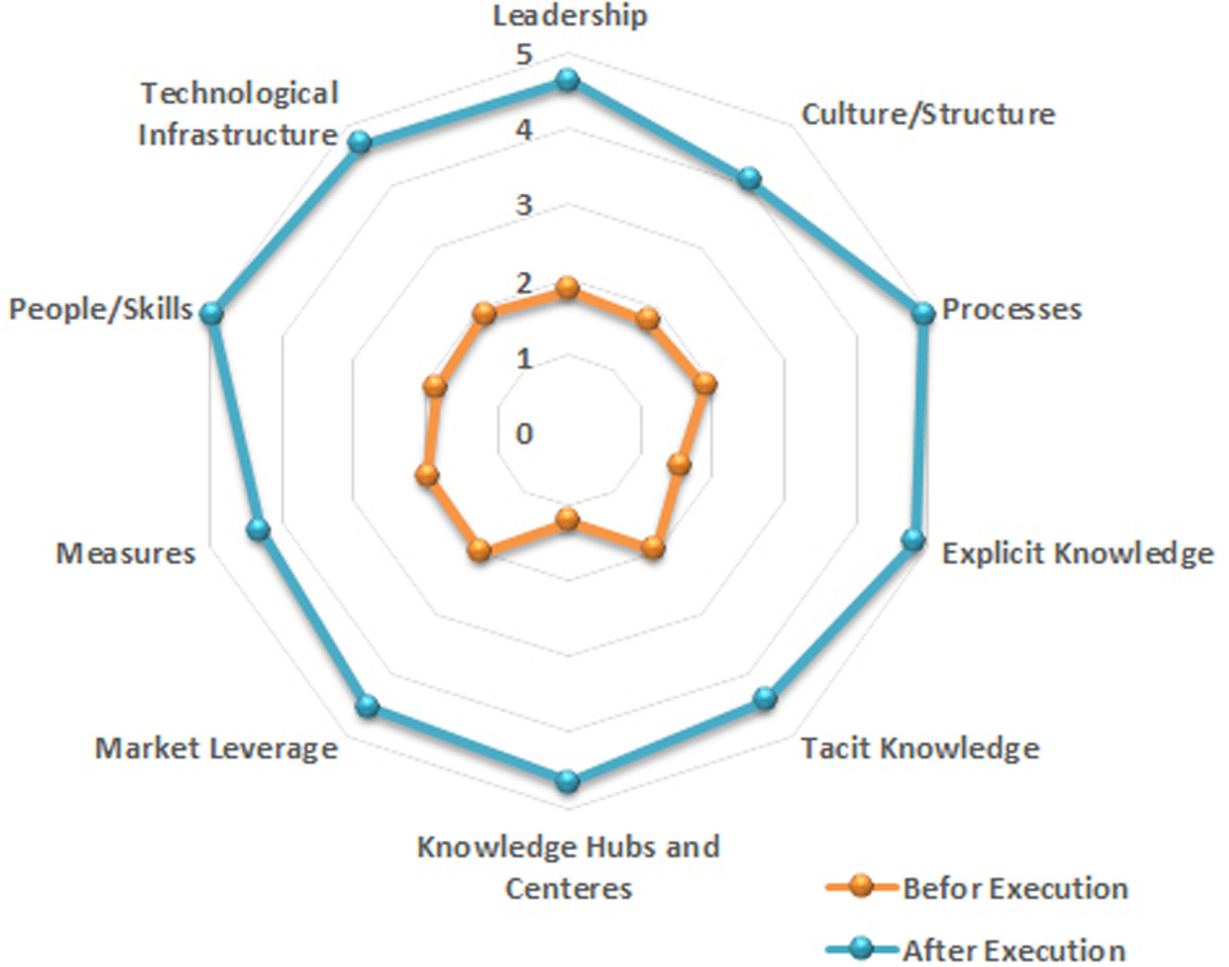
The scores of Skyrme metrics before and after implementation of our KA solution for the study organization.

Fig 7 indicates the radar diagram numerical values that are related to the ten metrics within the Skyrme assessment maturity model for the study organization before and after implementation of the KA plan by applying the proposed methodology and framework. These values were obtained using the questionnaires completed by experts in the field. The radar diagram indicates that the mean of scores for all ten metrics within the Skyrme maturity model achieved significant improvement after implementation of the proposed KA solution. This effect caused the mean of all scores for the metrics within the first and second levels prior to implementation to change to the fourth and fifth levels after implementation of the solution. Therefore, we can conclude that implementation of the proposed solution within knowledge environments produces improvement in the level of metrics, which are effective in organization knowledge management.

### 2. Assessment through qualitative methods

The precise assessment of frameworks, models and methodologies via quantitative methods is challenging because these products are descriptive instead of prescriptive. The conditions that override project teams and organizations are also effective in the manner in which these products are applied. Consequently, qualitative assessment methods are generally employed to assess the products obtained using applying qualitative research methods.

To evaluate the validity of our proposed solution, we are dependent on the viewpoints of experts in the field of KM. Fifty questionnaires were distributed to assess the viewpoints of experts in the of Management and Computer Science department at the University of Tehran, the Amirkabir University of Technology, and the Islamic Azad University in Tehran North Branch, who have written a significant number of papers related to KA and KM and have conducting numerous studies on these two subjects. These faculty members also work as knowledge managers of knowledge architecture in the R&D departments of organizations, such as the Islamic Republic of Iran Ports and Maritime Organization and Tehran Municipality. The results of thirty questionnaires were used for the data analysis. The selection of the survey questions was in accordance with the objectives and goals of this paper. To establish the reliability of the questionnaire, the Cronbach’s alpha method, which was shown to have a suitable rate (α = 0.827) by the SPSS software version 21, was employed; the Delphi technique was applied as the consensus methodology. As discussed in the next section, the statistical methods of standard deviation, mean and binomial test [34] were used to analyze the data.

The data collected from the questionnaires were initially analyzed using the method of standard deviation. A standard deviation less than 1 (0.702–0.610) indicates the acceptability of the data (Table 7).

**Table 7:** Results of standard deviation and mean.

	Mean	Std. Deviation	N
**Answer to question no. 1**	4.43	0.626	30
**Answer to question no. 2**	4.13	0.681	30
**Answer to question no. 3**	4.30	0.702	30
**Answer to question no. 4**	3.97	0.669	30
**Answer to question no. 5**	4.13	0.681	30
**Answer to question no. 6**	3.87	0.681	30
**Answer to question no. 7**	3.93	0.691	30
**Answer to question no. 8**	4.20	0.610	30
**Answer to question no. 9**	3.10	0.662	30
**Answer to question no. 10**	3.83	0.699	30

To compare the cores from the questionnaire with the highest score and the mean score, the score for each completed questionnaire was separately calculated. The mean of the scores for 30 questionnaires, which was higher than the mean score (30), was subsequently calculated (37). These results indicated that the proposed solution was considered to be satisfactory by experts.

With regard to the qualitative and descriptive nature of the hypothesis and the proposed solution in this paper, the Likert scale [35] was employed to attain the responses of the experts. The binomial test was also used to statistically analyze the results. The binomial test was administered for ten questions within the questionnaire:

1. The completeness and comprehensiveness of the approach
2. The correlation between the methodology and the supervising framework
3. The responsiveness of the approach toward the issue of KA within large-scale organizations
4. The details in the methodology
5. The comprehensiveness of the phases and the steps of the methodology
6. The ease of execution of the approach
7. The flexibility of the approach
8. The focus on the innate requirements of large-scale architecting by the approach
9. The superiority to other solutions
10. The flexibility of the service-oriented style for knowledge interoperation

In all questions, μ≤3 is interpreted as rejection of the problem and μ>3 signifies acceptance of the problem. The test was calculated by the SPSS software, version 21. Table 8 indicates the results of the test that was administered for the results of the questionnaires. The results of the binomial test emphasize that all questions possess a suitable validity by experts.

**Table 8:** Results of binomial test.

		Category	N	Observed Prop.	Test Prop.	Exact Sig. (One-tailed)
**Answer to question no. 1**	Group 1	< = 3	2	0.1	0.6	0.000^a^
	Group 2	>3	28	0.9		
	Total		30	1.0		
**Answer to question no. 2**	Group 1	< = 3	5	0.2	0.6	0.000^a^
	Group 2	>3	25	0.8		
	Total		30	1.0		
**Answer to question no. 3**	Group 1	< = 3	2	0.1	0.6	0.000^a^
	Group 2	>3	28	0.9		
	Total		30	1.0		
**Answer to question no. 4**	Group 1	< = 3	7	0.2	0.6	0.000^a^
	Group 2	>3	23	0.8		
	Total		30	1.0		
**Answer to question no. 5**	Group 1	< = 3	5	0.2	0.6	0.000^a^
	Group 2	>3	25	0.8		
	Total		30	1.0		
**Answer to question no. 6**	Group 1	< = 3	9	0.3	0.6	0.001^a^
	Group 2	>3	21	0.7		
	Total		30	1.0		
**Answer to question no. 7**	Group 1	< = 3	8	0.3	0.6	0.000^a^
	Group 2	>3	22	0.7		
	Total		30	1.0		
**Answer to question no. 8**	Group 1	< = 3	3	0.1	0.6	0.000^a^
	Group 2	>3	27	0.9		
	Total		30	1.0		
**Answer to question no. 9**	Group 1	< = 3	5	0.2	0.6	0.000^a^
	Group 2	>3	25	0.8		
	Total		30	1.0		
**Answer to question no. 10**	Group 1	< = 3	10	0.3	0.6	0.003^a^
	Group 2	>3	20	0.7		
	Total		30	1.0		

^a^Alternative hypothesis states that the proportion of cases in the first group < 0.6.

## Conclusion

The most important asset that causes organizations to become pioneers in a competitive world is the amount of knowledge at their disposal. KM can be considered to be the process of creating value from an organization’s intangible properties. Although many frameworks and methodologies have been proposed for implementing KM within enterprises, numerous enterprises have not successfully implemented their KM projects despite large monetary investments [2]. One method for increasing efficiency in KM within enterprises is a proper KA solution, which can assist an enterprise in the application of KM.

Significant deviations from classical architecting to large-scale architecting and the review of numerous KA solutions, such as Jafari [9] and Lusa [10] generated the following three limitations in conducting KA within large-scale organizations:

- Limited focus on large-scale architecting properties
- Lack of a KA development process with a suitable level of details
- Lack of a supervising KA framework

Regarding these limitations and the failure of the proposed solutions in this domain to sufficiently address these shortcomings, we attempted to develop a methodology and a supervising framework for conducting knowledge architecture within large scale organizations by merging existing concepts in the area of knowledge management and information architecture framework.

To achieve this goal, the basic concepts related to this subject were explained. The initial structure of the supervising framework was based on the information architectural framework of Zachman, which has been identified as the main framework in the information architecture field. By adding the architecture level dimension, service abstraction and changing the field of all cells within Zachman’s enterprise information architectural framework from information to knowledge, we obtained the ideal supervising KA framework. The initial structure of the methodology was derived from the information architectural methodology of EAP, which is the methodology that is most congruent with Zachman’s framework, and was updated by adding the iteration and evolution cycle and the project management layer, changing its phases, and reapplying the CommonKADS knowledge engineering methodology models to attain the desired KA methodology within large-scale organizations. The results related to the validity of our proposed solution, which was evaluated by performing a case study and assessing the viewpoints of some experts, emphasize the proposed solution.

The uniqueness of this work is threefold. Firstly, a methodology is proposed for establishing KA within large-scale organizations. This methodology gives valuable information and guidelines with a suitable level of details that can help knowledge architectures to accomplish KA through their organization successfully. Secondly, a supervising KA framework is presented as an initial structure for our proposed methodology. It is used to underpin and provide a set of theoretical bases and fundamental principles for performing the relevant actions and activities in the proposed KA methodology. Thirdly, the presented approach has greater applicability than any other solutions for conducting KA within large-scale organizations due to the fact that it has been designed based on large-scale architecting properties. Accordingly, the most important contributions of this study are as follows:

- To provide a methodology for establishing KA within large-scale organizations
- To provide a supervising KA framework within large-scale organizations
- KA is the reason for achieving success in a KM project within large-scale organizations
- A supervising framework, which considers the large-scale architecting properties and employs a suitable level of details, are the main features that must be addressed by a KA methodology within large-scale organizations

According to the results of this study, the following suggestions are proposed for future studies:

- Present KA frameworks and methodologies within large-scale organizations that have been developed based on other information architectural frameworks, such as TOGAF
- Confirm the validity of the proposed KA methodology using qualitative assessments
- Standardize some KA methodology models, which have not been obtained from CommonKADS methodology models, by applying a model-driven architectural framework
- Solicit foreign experts about the validity of this KA approach

## Supporting Information

S1 Appendix Research questionnaire. (DOCX)
