# Supplementary material for: Presenting an Approach for Conducting Knowledge Architecture within Large-Scale Organizations
Source: PLoS One. 2015 May 20;10(5):e0127005. doi: 10.1371/journal.pone.0127005 (PMC4438864; doi:10.1371/journal.pone.0127005)
Supplement: S1 Appendix — (DOCX) [file pone.0127005.s001.docx]

| **S1 Appendix. Research questionnaire** | | | | | | | |
| --- | --- | --- | --- | --- | --- | --- | --- |
| Personal Characteristics: | | | | | | | |
| Scientific Rank: Full professor □ Associate professor □ Assistant Professor □ | | | | | | | |
| Instructor Professor□ Manager □ | | | | | | | |
| Row | Question | Strongly Agree | | Agree | No Comment | Disagree | Strongly Disagree |
|  |  | (5) | | (4) | (3) | (2) | (1) |
| 1 | The approach is complete and comprehensive | |  |  |  |  |  |
| 2 | The methodology has complete correspondence with the supervising framework | |  |  |  |  |  |
| 3 | The approach responds to the issue of KA within large-scale organizations | |  |  |  |  |  |
| 4 | The level of detail in this methodology is sufficient | |  |  |  |  |  |
| 5 | The phases and steps of the methodology are complete and comprehensive | |  |  |  |  |  |
| 6 | The applicability and execution of the approach is easy | |  |  |  |  |  |
| 7 | The approach is flexible | |  |  |  |  |  |
| 8 | The approach addresses the innate requirements of large-scale architecting | |  |  |  |  |  |
| 9 | The approach is more appropriate than other solutions | |  |  |  |  |  |
| 10 | The service-oriented style is flexible for knowledge interoperation | |  |  |  |  |  |
